# Microclimate Modification, Evapotranspiration, Growth and Essential Oil Yield of Six Medicinal Plants Cultivated Beneath a Dynamic Agrivoltaic System in Southern Italy

**DOI:** 10.3390/plants14152428

**Published:** 2025-08-05

**Authors:** Grazia Disciglio, Antonio Stasi, Annalisa Tarantino, Laura Frabboni

**Affiliations:** Department of Agriculture, Food, Natural Resources and Engineering (DAFNE), University of Foggia, Via Napoli 25, 71122 Foggia, Italy; antonio.stasi@unifg.it (A.S.); annalisa.tarantino@unifg.it (A.T.); laura.frabboni@unifg.it (L.F.)

**Keywords:** dynamic agrivoltaic, medicinal crops, microclimatic factors, crop evapotranspiration, plant biomass, essential oils yield

## Abstract

This study, conducted in Southern Italy in 2023, investigated the effects of a dynamic agrivoltaics (AV) system on microclimate, water consumption, plant growth, and essential oil yield in six medicinal species: lavender (*Lavandula angustifolia* L. ‘Royal purple’), lemmon thyme (*Thymus citriodorus* (Pers.) Schreb. ar. ‘Aureus’), common thyme (*Thymus vulgaris* L.), rosemary (*Salvia rosmarinus* Spenn. ‘Severn seas’), mint (*Mentha spicata* L. ‘Moroccan’), and sage (*Salvia officinalis* L. subsp. *Officinalis*). Due to the rotating solar panels, two distinct ground zones were identified: a consistently shaded area under the panels (UP), and a partially shaded area between the panels (BP). These were compared to an adjacent full-sun control area (T). Microclimate parameters, including solar radiation, air and leaf infrared temperature, and soil temperature, were recorded throughout the cultivation season. Reference evapotranspiration (ET_O_) was calculated using Turc’s method, and crop evapotranspiration (ET_C_) was estimated with species-specific crop coefficients (K_C_). Results showed significantly lower microclimatic values in the UP plot compared to both BP and especially T, resulting in ET_C_ reductions of 81.1% in UP and 13.1% in BP relative to T, an advantage in water-scarce environments. Growth and yield responses varied among species and treatment plots. Except for mint, all species showed a significant reduction in fresh biomass (40.1% to 48.8%) under the high shading of UP compared to T. However, no biomass reductions were observed in BP. Notably, essential oil yields were higher in both UP and BP plots (0.60–2.63%) compared to the T plot (0.51–1.90%). These findings demonstrate that dynamic AV systems can enhance water use efficiency and essential oil yield, offering promising opportunities for sustainable, high-quality medicinal crop production in arid and semi-arid regions.

## 1. Introduction

Agrivoltaic (AV) systems are innovative mixed-use production systems that integrate crop cultivation with solar energy generation. In these systems, crops are grown beneath solar panels, which alters environmental conditions such as shade levels, temperature, humidity, and water availability [1]. Among these, the variation in microclimatic factors is one of the most critical considerations for effective agricultural practice under AV arrays [2]. Latitude and prevailing weather conditions significantly influence crop performance, particularly in regions with suboptimal climates for agriculture [3]. Variables such as solar radiation, temperature, humidity, wind, and precipitation can all impact plant growth [4]. While sunlight is vital for photosynthesis, excessive exposure can be detrimental. In fact, the photosynthetic light-response curve shows how a plant’s net photosynthesis changes with light intensity. The light compensation point is the minimum light level at which photosynthesis equals respiration, resulting in no net CO_2_ uptake. Below this point, respiration dominates, leading to a net carbon loss. At low light intensities, photosynthesis increases linearly with light. At higher intensities, it plateaus (reaching saturation). Excessively high light can damage the photosynthetic machinery [5]. High solar intensity and prolonged exposure increase transpiration rates as plants attempt to cool themselves. However, excessive light can also lead to photoinhibition—a reduction in photosynthesis caused by light-induced stress. This damage to plant tissues can significantly reduce yield [5,6], particularly when extreme heat coincides with sensitive phenological stages of plant development [7]. This can occur in arid and semi-arid regions, such as Mediterranean areas with high natural irradiation. Southern Italy, for example, faces increasing drought and heat stress due to its high solar irradiance. To mitigate excessive heat, shading is commonly used for some tree crops, employing shading nets or whitening treatments [8,9]. However, reducing solar radiation can decrease photosynthesis and plant productivity, even in summer [10]. On the other hand, shading can improve radiation use efficiency by increasing the proportion of diffuse irradiance [11]. Controlled shading provided by agrivoltaic (AV) systems can protect crops from excessive light and heat stress, thereby enhancing their resilience to climate variability [12,13]. In fact, the primary environmental change under an AV canopy is reduced solar radiation, which has been shown to improve plant performance [14]. However, other microclimate factors, such as air, leaf, and soil temperature, humidity, as well as photosynthetically active radiation (PAR) are also modified [15]. A study has demonstrated that shading by photovoltaic panels can reduce leaf temperature by approximately 5 °C and increase relative humidity by about 10%, creating more favorable growing conditions [16].

The extent of irradiance reduction is influenced by several factors, including the panel configuration (e.g., tilt angle, orientation, and elevation), the spacing between panel rows, and temporal variations such as time of day and season. Empirical evidence from agrivoltaic research indicates that solar irradiance under fixed-tilt panels is typically reduced by 30–60%, with more densely arranged arrays potentially reducing irradiance by up to 80%. However, systems using elevated or tracking panel configurations, such as those used in our research, tend to allow for greater light penetration, with irradiance reductions in the 10–40% range [17].

This dual function of solar panels, not only generating electricity but also moderating extreme microclimatic conditions, makes them especially valuable in water-limited environments. The shading reduces evapotranspiration rates, contributing to water conservation and fostering a more stable and productive microclimate for agriculture [18]. Indeed, by limiting direct solar exposure and reducing surface evaporation, solar panels can contribute to higher soil moisture retention. Several studies have shown that soils beneath PV panels tend to stay wetter for longer periods following precipitation or irrigation events [2]. This increased retention can have significant implications for irrigation efficiency and plant water use, particularly in regions facing water scarcity. Moreover, the distribution of water in the soil profile may change under panels, as the reduced surface evaporation may encourage deeper infiltration and reduce crust formation, potentially enhancing root access to moisture [19].

Due to the impact of solar panels on agricultural systems, numerous studies have been carried out to assess crop productivity under their influence [2,18,20,21,22,23,24,25,26,27]. These researchers have shown that AV systems, integrating photovoltaic panels with crop cultivation, can have both positive and negative effects on plant growth and quality. These outcomes depend on several factors, including crop type, panel mounting configuration, and local climatic conditions. Some crops are naturally more shade-tolerant than others. Leafy vegetables such as spinach, arugula, celeriac, and lettuce, as well as crops like broccoli, tomatoes, and potatoes, often benefit from partial shading. In contrast, crops like corn and wheat typically require high levels of sunlight and perform poorly under reduced light conditions. However, excessive light and drought stress negatively impact all crop types. Studies have indicated that most horticultural crops show no significant changes in growth or quality when cultivated under AV systems with a panel cover ratio of 25% or less, with average yield reductions of under 25%. In contrast, panel coverage between 50% and 100% generally results in growth inhibition, with the notable exception of strawberries and spinach, which demonstrated improved yields at 50% and 60% coverage, respectively [28]. Therefore, selecting shade-tolerant crops is critical for the successful implementation of AV systems. Identifying and cultivating species that thrive under partial shade can support the wider adoption of agrovoltaics while sustaining or even enhancing agricultural productivity [29]. In 2024, the medicinal herb sector covered approximately 7300 hectares, producing 4000 tons of dry material and 350 tons of essential oils. Over 67% of this area is located in the southern regions (Sicily, Puglia, Sardinia) [30].

In Southern Italy, various medicinal and aromatic plants are cultivated primarily for their essential oils, which are widely used in pharmaceuticals, cosmetics, and aromatherapy. The region’s Mediterranean climate, characterized by hot, dry summers, abundant sunlight, and low humidity, can induce physiological stress in these plants, affecting both biomass production and essential oil yield. While these conditions often enhance the synthesis of secondary metabolites, including essential oils, prolonged droughts may negatively impact certain species by reducing biomass and oil output. A detailed understanding of species-specific responses to environmental stressors is therefore essential. Optimized cultivation strategies, such as agrivoltaic (AV) systems, offer a promising solution. However, the performance of medicinal herbs under AV conditions, particularly in terms of water use, biomass accumulation, and essential oil content, remains poorly studied. Different species may respond differently to the microclimatic changes induced by AV systems, making species-specific insights critical. This research aims to evaluate the impact of solar radiation, air, plant, and soil temperature—measured both under photovoltaic panels and in full sun—on evapotranspiration, plant growth, and essential oil yield in six medicinal crops: lavender, lemon thyme, common thyme, rosemary, mint, and sage.

## 2. Materials and Methods

### 2.1. Site and Field Experiment Design

**Figure 1:**
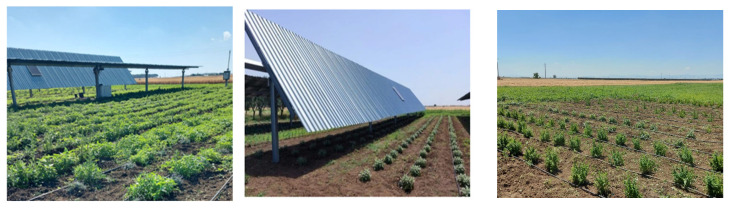
The support structures arranged in a north–south direction in parallel rows, and the adjacent open-field area. This structure is a part of AV system, and is located in the agricultural area of San Severo (41°41 N, 15°22 E; 86 m a.s.l.) in the Foggia district B (Fotos: Frabboni L.).

The study was conducted during the 2023 growing season at the Dynamic Agrivoltaic System (AV) field operated by M2 Energy S.r.l., located in the agricultural area of San Severo (41°41 N, 15°22 E; 86 m a.s.l.) in the Foggia district. The structural features of the AV system and the soil’s physical and chemical properties were previously described by Disciglio [31]. The solar panel array, aligned north–south, consisted of panels measuring 4.166 m in width and spaced 8.85 m apart. When projected onto a horizontal plane, the panels covered 47% of the ground surface. The system allows the panels to rotate up to approximately 45 degrees along an east–west axis to track the Sun’s daily movement (Figure 1). As a result of this rotation, distinct ground zones form beneath the panels: a consistently shaded area (UP), and an intermittently shaded area between the panels (BP). These were compared with an adjacent area exposed to full sun (T). Six aromatic crop species were cultivated in each of these zones to evaluate the effects of varying light conditions.

**Figure 2:**
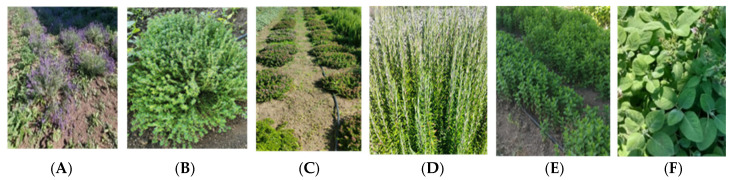
Medicinal plants (from left to right): (**A**) (*Lavandula angustifolia* L. ‘Royal purple’), (**B**) lemmon thyme (*Thymus citriodorus* (Pers.) Schreb. ar. ‘Aureus’), (**C**) common thyme (*Thymus vulgaris* L.), (**D**) rosemary (*Salvia rosmarinus* Spenn. ‘Severn seas’), (**E**) mint (*Mentha spicata* L. ‘Moroccan’), and (**F**) sage (*Salvia officinalis* L. subsp. *Officinalis*) (Fotos: Frabboni L.).

This study investigated the cultivation of six perennial medicinal species (Figure 2), including lavender (*Lavandula angustifolia* L. ‘Royal purple’), lemmon thyme (*Thymus citriodorus* (Pers.) Schreb. ar. ‘Aureus’), common thyme (*Thymus vulgaris* L.), rosemary (*Salvia rosmarinus* Spenn. ‘Severn seas’), mint (*Mentha spicata* L. ‘Moroccan’), and sage (*Salvia officinalis* L. subsp. *Officinalis*) in comparison among the Up, Bp and T areas. In each area, the medicinal crops were transplanted in continuous rows with 1 m spacing between plants (1 × 1 m) in May 2022. As these are perennial plants capable of remaining in the soil for several years due to their basal buds, this study examined their cultivation in 2023, the second year after planting.

The field trial covered an area of 30 × 115 m (3450 m^2^), with half under the AV system (1725 m^2^) and the other half in full sun conditions in the T zone (1725 m^2^). The region under the AV system was further divided into UP and BP zones (each measuring 862 square meters).

Of the entire field, the experimental part consisted of six agrivoltaic panels, each 30 m long, spaced 8.85 m apart. Each of the six aromatic plant species was grown in rows within the area under each panel—half in the fully shaded section (UP), and half in the alternately shaded section (BP)—and extended for another 30 m beyond the panel into the adjacent open-field area (T).

The experiment follows a single-factor design (light treatment), with independent replication within each species. Species are treated as separate experimental groups and are not statistically compared, thus excluding a factorial or blocked design structure.

### 2.2. Cultural Practices

The agricultural management practices applied to the medicinal crops during the experimental trial were those commonly used by farmers in organic farming systems. Organic nitrogen fertilization was carried out at the beginning of the vegetative growth phase. Weed control was performed mechanically through soil tillage between the rows. Drip irrigation lines (with 2 L h^−1^ drippers spaced 40 cm apart) were laid on the ground along the tree rows, delivering an average of 1500 m^3^ ha^−1^ of water during the growing season. Pests generally do not pose a significant problem in organic systems, as healthy plants grown in well-balanced, nutrient-rich soil are better able to resist pest and disease attacks.

### 2.3. The Climate

The site of the research was in a typical semi-arid zone, characterized by a Mediterranean climate, classified as an “accentuated thermomediterranean” [32] with temperature that may fall below 0 °C in winter and exceed 40 °C in summer, and rainfall mostly concentrated in the winter months with a long-term annual average of 559 mm [33].

During the growing cycles of medicinal crops, from April to August 2023, daily climate data, including maximum and minimum temperatures and air humidity, radiation, wend speed and total precipitation, were recorded at the nearest meteorological station, which was a few kilometers from the experimental area, and supplied by the Centro Funzionale Decentrato Regione Puglia [34].

Table 1 presents the monthly climate data recorded during the growing season of medicinal crops, from April to August 2023. The seasonal average values for key weather parameters were as follows: maximum temperature 29.8 °C, minimum temperature 15.4 °C, relative air humidity 88.2%, radiation 249.6 W·m^−2^, wind speed 3.5 m·s^−1^, and total precipitation 51.3 mm. Notably, the average maximum and minimum temperatures during the growing season (29.8 °C and 15.4 °C, respectively) were higher than the historical multi-year averages (26.3 °C and 14.9 °C, respectively) for the same period, as reported by Rubino et al. (2015) [35] for the years 1951–2001. This rise in temperature reflects a broader trend consistent with ongoing climate change. Additionally, a distinguishing climatic characteristic of the experimental area is the high wind speed, with a seasonal average of 3.6 m·s−1.

### 2.4. Microclimate Measurements

Microclimatic parameters such as solar radiation, air and soil temperature, and infrared leaf temperature were measured during crop growth in the UP, BP, and T plots. These parameters were recorded during daylight hours every two hours (from 7:00 AM to 6:00 PM) to capture different solar elevation angles and radiation levels on the canopy. Measurements were taken at approximately 10-day intervals throughout the crop cycle, starting on April 10th and continuing until August, on representative days. The measurements were manually recorded using the following instruments: a solar power meter (TES, Taipei City, China, model TES 1333, Acc = ±0.9%), an air thermometer (TorAnn S.A.S Strumenti, Bari, Italy, model HD 2328.0), an infrared thermometer (Lafayette, model TRI-88, Tokyo, Japan, Acc. = ±1%), and a soil thermometer (EXTECH Instruments, model MO750-EU-IT, Taipei City, China, Acc = ±0.9%). Solar radiation and air temperature were measured at a height of 2 m above the ground, while soil temperature was measured at a depth of 10 cm between plant rows. The infrared thermometer was manually positioned at a 30-degree angle toward the area with the densest canopy, with measurements taken every meter. It should be noted that microclimatic measurements on each day did not last for 2 h. Instead, data collection was completed rapidly, within 10–15 min for all treatments, as it was carried out simultaneously by four operators, each responsible for measuring a single microclimatic parameter. Each measurement was replicated three times, specifically in the two central rows located in the middle of each plot. Infrared leaf temperature measurements were taken on both the east and west sides of the canopy, with six measurements per side, per species, and per hour.

### 2.5. Water Consumption: Theoretical Calculation of the ET_0_ and Etc

Considering that crop evapotranspiration primarily occurs during daylight hours, monthly average daytime microclimatic data (specifically solar radiation and air temperature) were used to calculate the reference evapotranspiration (ET_0_) for each area (UP, BP, T), employing Turc’s empirical method [36], which is suitable for humid climates (relative humidity > 50%). Crop evapotranspiration (ETc) was then determined by multiplying ET_0_ by the crop coefficient (Kc), which varies based on crop type and growth stage [37].

However, studies conducted in southern Italian environments have shown that the Turc formula generally overestimates ET_0_ by approximately 20% when compared to values obtained through weighing lysimeters [38]. This overestimation is attributed to the Turc equation’s greater sensitivity to air temperature than to solar radiation [39]. To mitigate this bias, a correction factor of c = 0.80 was applied to the original formula. The corrected version of the Turc equation, as proposed by Calcagno [39], is expressed as follows:

$$\mathrm{For}\;\mathrm{RH}\;>\;50\%\;{\mathrm{ET}}_{0}=\;(c)\;0.013\;\frac{T\;mean}{T\;mean\;+\;15}\;(23.885Rad\;+\;50)$$

where

*ET_0_ * represents the reference evapotranspiration, expressed in mm/day, averaged over monthly periods;
*T* = monthly mean daytime air temperature in °C in °C;
*Rad* = monthly mean daytime solar radiation in Wm^−2^;
(*c*) = local correction factor = 0.80.

The crop evapotranspiration (ETc) was calculated monthly by multiplying the reference evapotranspiration (ET_0_) by the crop coefficient (Kc) and the number of days in the month, following the method proposed by Allen et al. [40]. Standard indicative values of Kc for medicinal crops, as reported in various studies [41,42,43,44,45], range from 0.40 during the initial vegetative stage to 0.95 during the full productive growth stage. Fortnightly values of ET_0_ and Kc were used starting from the basal stage of the plant in April (when irrigation also begins) until the harvest stage in August.

In summary: *ETc* = *ET_0_* × *Kc*

where

*ET_0_* is the reference evapotranspiration, expressed in mm/day, averaged over monthly periods;
*Kc* is the crop coefficient, which varies depending on crop type and growth stage;
*ETc* is the crop evapotranspiration expressed in mm/day, averaged over monthly periods.

### 2.6. Plant Growth Measurements

At the time of harvest during the balsamic phase in July 2023, growth and yield measurements were collected from three representative plants in each experimental plot (UP, BP, and T). Plant height was measured from ground level (considered as 0), and plant width was measured along the east–west axis using a tape measure; all measurements were recorded in centimetres. To assess biomass production, the entire crop was manually harvested by cutting the plants at soil level. The fresh weight of each plant was immediately recorded. A representative subsample of the aerial biomass was oven-dried at 35 °C until it reached a constant weight, in order to prevent thermal degradation of volatile compounds, such as essential oils. The dry weight (DW) was then measured. Essential oil yield was determined from the dried leaves and expressed as a percentage (*w*/*w*) relative to the sample’s dry weight. Oil extraction was performed via steam distillation using a 62 L stainless steel extractor (Albrigi Luigi EO 131, Verona, Italy), following the method described by Frabboni et al. [46].

### 2.7. Statistical Analysis

The results were analysed using one-way analysis of variance (ANOVA) with the JMP software package (version 14.3; SAS Institute Inc., Cary, NC, USA). Mean comparisons were performed using Tukey’s post hoc test, with statistical significance set at *p* < 0.05. Percentage data were arcsine-transformed prior to ANOVA to stabilize variance.

## 3. Results

### 3.1. Effect of AV System on Microclimate

The presence of panels spatially altered diurnal microclimate parameters, such as solar radiation, air temperature, foliar infrared temperature, and soil temperature.

**Figure 3:**
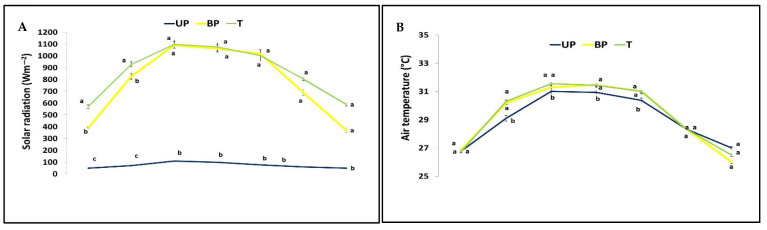

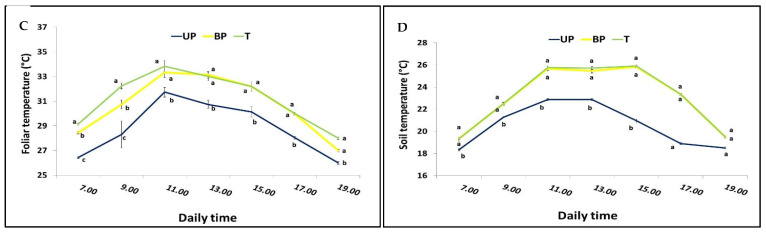
Diurnal trends of solar radiation (**A**), air temperature (**B**), foliar infrared temperature (**C**), and soil temperature (**D**) measured under the panels (UP), between the panels (BP), and in the open air (T) during daytime hours. Measurements were taken every two hours (from 7:00 AM to 7:00 PM) on several representative days throughout the 2023 growth cycle of the medicinal plants. Data are expressed as means ± SD. Different letters indicate significant differences among treatments, whereas the same letters denote non-significant differences, based on Tukey’s test (*p* ≤ 0.05).

Figure 3 shows the average seasonal trends of these daytime microclimate parameters, measured every 2 h over several days during the cropping season in the compared cultivation areas: UP, BP, and T.

The seasonal daytime solar radiation, measured at 2 h intervals, showed significant differences between the compared areas. The values in the UP plot were significantly lower (*p* ≤ 0.05), ranging from 50 to 110 Wm^−2^ during the day, whereas the BP plot recorded higher readings, between 388 and 1091 Wm^−2^. The T plot, in turn, exhibited even greater variation, with values ranging from 570 to 1099 Wm^−2^.

Notably, solar radiation in the BP area peaked at noon when the panels were in a horizontal position, reaching its highest value (1063 Wm^−2^), similar to the T plot’s reading (1075 Wm^−2^). However, during other hours of the day, when the panels rotated and experienced partial shading or limited sun exposure, the radiation levels were lower.

Daytime air temperature showed minimal differences between the BP and T areas. However, unlike solar radiation, which decreased significantly (*p ≤* 0.05) in both UP and BP compared to the T area, only UP exhibited a statistically significant reduction in temperature relative to T, particularly during midday. Consequently, the median daily mean temperature was slightly lower in UP (29.0 °C) than in BP (29.3 °C) and notably lower than in T (29.5 °C)

Infrared leaf temperature measurements taken during the day revealed minimal differences among the medicinal species but significant variation across treatments. The UP plot consistently recorded the lowest temperatures, ranging from 26.1 °C to 32.2 °C. In contrast, the BP and T plots exhibited only slight differences, with temperatures ranging from 30.0 °C to 35.1 °C in the BP plot and 30.1 °C to 38.9 °C in the T plot. Both the BP and T plots displayed distinct diurnal patterns, with the most pronounced differences occurring in the morning hours. During this time, the BP plot showed lower temperatures values, ranging from 0.5 °C to 1.5 °C lower, than the T plot.

**Figure 4:**
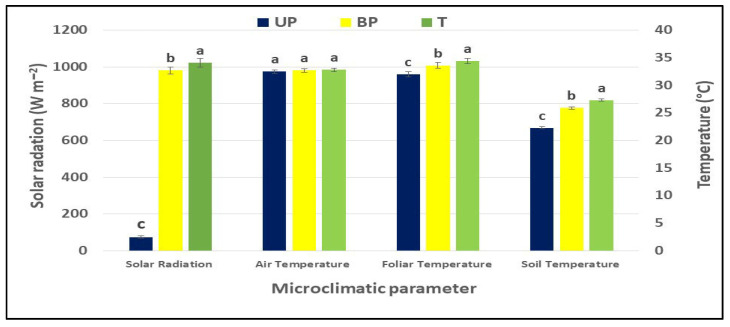
Mean daily (diurnal) microclimatic parameters—including solar radiation, air temperature, foliar infrared temperature, and soil temperature—measured in the cultivation areas under the different treatment plots: UP (under the solar panels), BP (between panels), and T (full-sun control area). Data are expressed as means ± SD. For each microclimatic parameter, columns labeled with different letters indicate statistically significant differences, whereas the same letters denote non-significant differences, based on Tukey’s test (*p* ≤ 0.05).

Soil temperatures were significantly lower in the UP plot, ranging from 18.3 to 22.9 °C, compared to the BP and T plots, which showed no statistically significant differences and ranged from 19.3 to 25.9 °C. This temperature difference is attributed to reduced solar radiation in the shaded areas beneath the panels, creating a cooler microclimate. Pronounced diurnal variations were observed, with the daily maximum soil temperature consistently lower in the UP treatment, particularly during the afternoon (Figure 4).

As shown in Figure 3, significant differences were observed in the mean daily (diurnal) microclimatic parameters over a seasonal period among the compared treatments. Lower values were found in the UP plot compared to the BP plot, which in turn were lower than those in the T plots for solar radiation (78.0 W m^−2^ vs. 981 W m^−2^ and 1022 W m^−2^, respectively), foliar infrared temperature (32.0 °C vs. 33.7 °C and 34.4 °C), and soil temperature (22.3 °C vs. 25.9 °C and 27.3 °C). These differences were statistically significant. In contrast, no significant differences were found in the daily mean air temperature (32.7 °C) across the plots, although the UP plot tended to be slightly cooler—by 0.2 °C and 0.3 °C compared to the BP and T plots, respectively.

### 3.2. Effect of AV System on Crop Water Use

Solar radiation and air temperature data recorded during daylight hours on representative days of the growing season (April to August 2023) were used to calculate the monthly average reference evapotranspiration (ET_0_) in the three areas—Up, BP, and T. The corrected Turc method was applied for this calculation. By multiplying the ET_0_ values by the crop coefficients (Kc), the monthly crop evapotranspiration (ETc) was determined, as presented in Table 2.

As expected, all these parameters increased throughout the season as the plant canopy developed, peaking in July. Mean seasonal air temperatures varied slightly among the plots, with values of 24.7 °C in UP, 25.0 °C in BP, and 25.1 °C in T. In contrast, mean seasonal solar radiation differed significantly, with the UP plot recording a notably lower value (85 W m^−2^) compared to BP (586 W m^−2^) and especially T (662 W m^−2^). Consequently, significant differences were also observed in both ET_0_ (reference evapotranspiration) and ETc (crop evapotranspiration). The total seasonal ET_0_ values were 281.2 mm for UP, 461.6 mm for BP, and 529.4 mm for T, while ETc totals were 78.9 mm (UP), 362.2 mm (BP), and 416.9 mm (T).

### 3.3. Effect of AV System on Plant Growth

**Figure 5:**
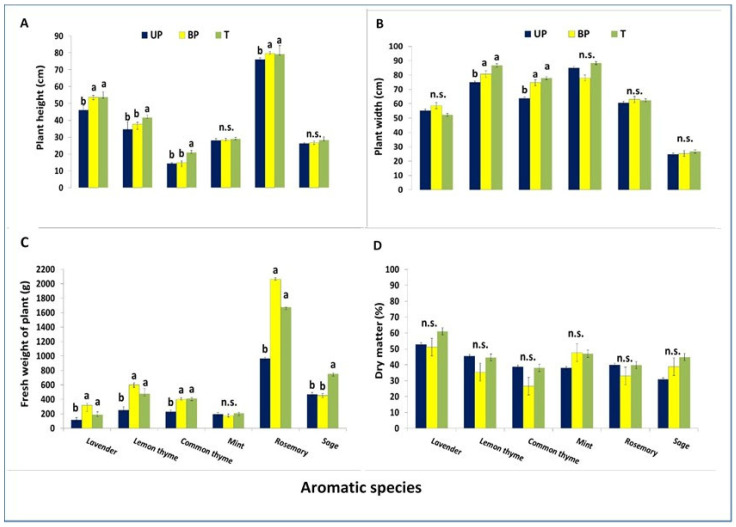
Average height (**A**), width (**B**), fresh weight (**C**), and dry matter content (**D**) of plants at harvest in 2023 under solar panels (UP), between panels (BP), and in open air (control—T). Data are presented as means ± standard errors from six replicates (n = 6). Within each medicinal species, means in the same column followed by different letters are significantly different according to Tukey’s test (*p* ≤ 0.05). Differences marked as n.s. indicate non-significance.

Regarding the comparison among treatments, plants of lavender, lemon thyme, and common thyme grown in UP showed significantly lower plant height (46 ± 3, 35 ± 5, and 14 ± 1 cm, respectively) and fresh weight (116 ± 29 250 ± 50, and 229 ± 23 g, respectively) compared to those in the T treatment (plant heights of 54 ± 3, 42 ± 2, and 21 ± 1 cm, respectively, and fresh weights of 190 ± 38, 476 ± 71, and 413 ± 15 g, respectively). Rosemary showed no difference in plant height (on average, 78± 4 cm) but had significantly lower fresh weight in UP (966 ± 14 g) than in BP (2060 ± 13 g) and T (1674 ± 3 g). Sage had significantly lower fresh weight in both UP (468 ± 30 g) and BP (452 ± 26 g) than in T (753 ± 14 g). Additionally, lemon thyme and common thyme under UP also exhibited smaller plant width (75 ± 10 and 64 ± 4 cm, respectively) compared to BP (81 ± 5 and 74 ± 3 cm) and T (87 ± 3 and 78 ± 3 cm). In contrast, mint showed no significant variation in morphological parameters across the three treatments. Finally, no difference was observed in dry matter among the three compared growing areas (Figure 5).

**Figure 6:**
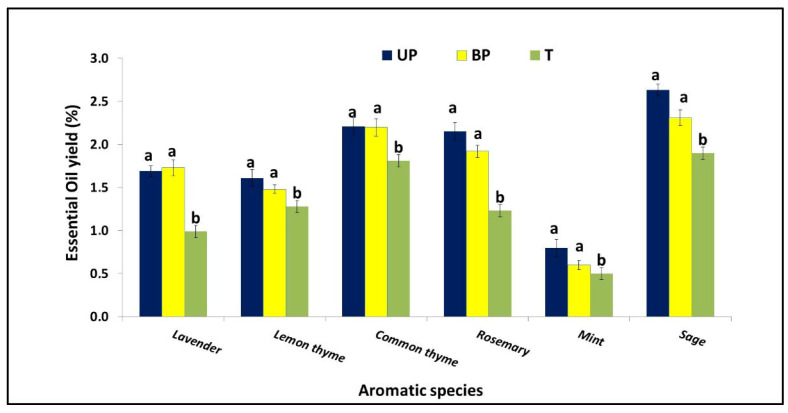
Mean essential oil yield (%) of aromatic crops compared across subpanels (UP), between panels (BP), and open-field plots (T). Data are expressed as means ± SD of six samples. For each medicinal species, columns labeled with different letters indicate statistically significant differences, while the same letters denote non-significant differences, based on Tukey’s test (*p* ≤ 0.05).

As shown in Figure 6, the yield percentage of essential oils (EOs) varied significantly among the studied medicinal crops and between different treatments. Common thyme, rosemary, and sage exhibited the highest average EO yields, ranging from 1.23% to 2.63%. Lavender followed with yields between 0.99% and 1.69%, while mint had the lowest EO content, ranging from 0.51% to 0.80%. Across all species, the UP and BP plots showed significantly higher average EO yields (0.60–2.63%) compared to the T plot (0.51–1.90%).

## 4. Discussion

### 4.1. Microclimatic Parameter and Plant Growth Variations

#### 4.1.1. Solar Radiation

The reduction in total solar radiation beneath the solar panels (UP) and in the alternately shaded areas between panels (BP) as the panels rotate—caused by shading—represents the most significant microclimatic variation. This, in turn, influences all other environmental parameters [47,48].

In this study, solar radiation was significantly reduced under the upper panels (UPs), with an 84.1% decrease relative to total radiation (T), while the reduction in the area between the panels (BP) was 12.9%. The distribution of sunlight and shade within agrivoltaic (AV) systems is strongly influenced by the configuration of the photovoltaic (PV) panels and is characterized by both temporal variability and spatial heterogeneity, especially in the BP area, where intermittent shading occurs due to the movement and rotation of the PV panels [20].

In other studies, a spacing of 3.2 m between solar panel rows resulted in approximately a 30% reduction in solar radiation [49,50]. Under a 35% coverage agrivoltaic (AgriPV) system, photosynthetically active radiation (PAR) was found to decrease by 70%. Additional research on agrivoltaic (AV) systems has reported reductions in solar radiation ranging from 30% to 70%, depending on system configuration and coverage [51].

#### 4.1.2. Daytime Air Temperature

Regarding daytime air temperature variations, our data differ from previous studies on legumes and grasses [47,52] and on different orchards [20,49,50,51], which reported significant reductions—up to 4 °C—in daily temperature fluctuations under shaded areas. In contrast, our study observed a considerably smaller reduction, ranging only from 0.3 to 0.5 °C. These discrepancies can be attributed to differences in climatic [53,54] agronomic [55], and structural conditions [56]. In particular, wind speed plays a critical role in influencing air temperature beneath agrivoltaic panels. Under high wind speed conditions—such as those in our study area (see Section 2.4)—the air temperature under the panels (UP) was not significantly affected, likely due to sufficient air circulation beneath the open structure [20].

#### 4.1.3. Infrared Plant Temperature

In this study, the average infrared leaf temperature was lower in the UP (under panel) area compared to both BP (between panel) and T (full-sun) conditions. Additionally, both the BP and T plots displayed distinct diurnal patterns, with the BP plot showing notably lower values than the T plot during the morning hours. This difference is likely due to shading from the low solar angle, which reduced solar exposure in the BP plot.

Given that leaf temperature, plant water status, and transpiration are closely linked, the cooler leaf temperatures observed under photovoltaics may reduce crop water demand [57].

The temperature difference (∆T) between the canopy infrared temperature and the surrounding air temperature can be used to compute the Crop Water Stress Index (CWSI), which estimates the relative transpiration rate at the time of measurement [58,59]. In this study, medicinal plants under the panels (UP) exhibited, on average, lower diurnal ∆T values than those in full sun (T). This suggests that UP plants may have lower CWSI values, indicating a reduced relative transpiration rate [60].

Furthermore, it should be noted that daily leaf temperature measurements revealed significant variability within a single plant’s canopy, particularly between sunlit and shaded leaves. Sun-exposed leaves often reached temperatures up to 5.5 °C higher than the surrounding air, especially during early morning and late afternoon, due to absorbed solar radiation [53]. In contrast, shaded leaves maintained more stable temperatures, typically close to or slightly below ambient air temperature [61]. Overall, leaf temperature did not directly correlate with air temperature (Tₐ) [62].

#### 4.1.4. Soil Temperature

On average, the median daily mean soil temperature in the UP plot was approximately 2.5 °C lower than in the BP and T treatments. These results align with recent findings from other studies [15,41,42], which reported that agrivoltaic (AV) systems reduce soil temperatures by 1–4 °C and lower water demand, benefiting crops in arid and semi-arid regions. By moderating temperature extremes, such systems can significantly improve growing conditions, particularly where high surface temperatures often stress plants and accelerate soil moisture loss. Cooler soil temperatures help maintain a more stable root zone environment, reducing evaporation and conserving soil moisture. This, in turn, decreases the overall water demand of crops, making irrigation more efficient and less frequent. These improvements enhance plant resilience to heat stress and support better nutrient uptake, root development, and yield stability under challenging climatic conditions [63].

#### 4.1.5. Crop Evapotranspiration

The observed reduction in solar radiation and lower air temperatures in both the UP and BP plots led to a notable decrease in crop evapotranspiration (ETc) compared to the T plots. During the crop season, average monthly ETc values showed a substantial overall reduction of 81.1% in the UP area and 13.1% in the BP area relative to the T plot. These findings are particularly relevant for water-scarce regions such as Puglia, as they indicate a significant decrease in atmospheric water demand for crops. Similar trends in reduced crop water requirements have been reported by Schweiger and Pataczek [25]. Additionally, model simulations for arid and semi-arid environments have predicted a 30–40% reduction in crop water consumption under static array configurations with 50% shading [64]. Furthermore, microclimatic alterations have been shown to lower water consumption needs by 6–31% [2].

#### 4.1.6. Plant Growth

Elevated light restriction in the under-panel (UP) area significantly reduced the growth of lavender, lemon thyme, common thyme, and rosemary, lowering plant height by 13.5%, 16.8%, 33.3%, and 4.6%, and fresh weight by 40.0%, 48.8%, 48.7%, and 40.6%, respectively, compared to the control (T). Sage showed a significant reduction only in fresh weight (42.4%). Lemon thyme and common thyme also had decreased plant width under UP (11.9% and 15.8%). Under the between-panel (BP) treatment, lemon thyme and common thyme saw height reductions of 23.8% and 32.2%. Mint showed no significant changes across treatments. Dry matter content remained stable across all species and treatments, indicating no effect from light availability.

These findings suggest that different medicinal plant species have distinct light requirements. In general, significant biomass reductions occurred under ultra-low light conditions (UP treatment), while partial shading (BP) did not produce the same extent of reduction, relative to full sunlight (T). Similar trends in harvestable crop biomass reductions due to light restriction have been reported by Reeza et al. [65], with yield reductions of 18% in okra, 50% in eggplant and Brazilian spinach, 70% in Chinese cabbage, and 90% in both green spinach and Chinese kale. The authors of [2] also observed yield reductions in crops such as maize and kiwifruit under shaded conditions.

However, since agrivoltaic (AV) systems are designed as co-productive systems that integrate energy generation and agricultural production, crop yield should not be evaluated in isolation. Instead, yield should be assessed alongside energy output and potential improvements in land-use efficiency to comprehensively evaluate AV system performance [66].

#### 4.1.7. Essential Oil Yield Content

Medicinal crops commonly cultivated in Southern Italy are highly valued for their essential oils, which are widely used in pharmaceuticals, cosmetics, and aromatherapy. The essential oil yields of all the medicinal crops studied were significantly higher in both the UP and BP plots (ranging from 1.8% to 2.5%) compared to the T plot (1.2% to 2.1%). The improved performance observed in crops grown within the agrivoltaic (AV) system may be attributed to the shading effect of the solar panels. This shading led to lower solar radiation and reduced air temperatures, which, along with a decreased presence of weeds—as previously reported by Disciglio et al. [31]—likely had a positive impact on crop development and essential oil production. The dynamic agrivoltaic system can improve the essential oil yield even when some land area is occupied by solar panels. The enhancement in essential oil content under shaded conditions is likely due to several physiological mechanisms, such as reduced photooxidative stress, altered secondary metabolite pathways, and improved microclimatic conditions (e.g., lower temperatures and moderated light intensity), which can stimulate the biosynthesis and accumulation of volatile compounds [67,68]. The medicinal crops, commonly cultivated in Southern Italy, are valued for their essential oils used in pharmaceuticals, cosmetics, and aromatherapy.

## 5. Conclusions

The integration of dynamic agrivoltaic (AV) systems in Southern Italy represents a promising synergy between renewable energy production and sustainable agriculture. While plant growth and biomass yield of medicinal crops—commonly cultivated in the region—respond variably to the microclimatic changes induced by AV systems, these systems can enhance the yield of essential oils. These oils are highly valued in the pharmaceutical, cosmetic, and aromatherapy industries.

Furthermore, since medicinal herbs, like all horticultural species, require adequate water supply, the reduced evapotranspiration rates under AV systems can be particularly beneficial in water-scarce areas. This highlights the importance of implementing effective water conservation strategies alongside AV deployment.

Although AV systems may lead to a decrease in biomass yield, they offer significant advantages in terms of increased essential oil production and reduced water consumption. Considering, in our case, the limited loss of cultivated land due to the support structures in the dynamic agrivoltaic system—approximately 1% of the total surface area, with a row spacing of 8.85 m and an average footprint of the supports—and taking into account an estimated 30% reduction in crop yield due to shading (in both UP and BP configurations), the potential gains in terms of efficiency, higher income from an estimated 30% increase in oil yield, and energy production from the system could lead to higher overall farm net returns.

However, to fully understand their viability, future research should focus on quantifying the economic trade-offs and evaluating the cost–benefit relationship of agrivoltaic systems in medicinal plant cultivation.

## Figures and Tables

**Table 1:** Monthly mean maximum and minimum temperatures (Tmax, Tmin), maximum and minimum relative humidity (RHmax, RHmin), solar radiation (Rad), wind speed (WS), and total precipitation (P) during the 2023 season.

Month	T_max_	T_min_	RH_max_	RH_min_	Rad	Ws	P
	(°C)	(°C)	(%)	(%)	(W·m^−2^)	(m·s^−1^)	(mm)
April	19.6	6.3	95.8	49.6	194	3.5	20.8
May	28.5	12.7	93.7	43.4	207	3.2	22.2
June	33.9	18.6	83.8	36.0	280	3.7	51.3
July	34.7	19.0	82.5	33.5	300	3.4	49.8
August	32.3	19.1	85.3	36.7	267	3.7	20.0
Mean	29.8	15.4	88.2	39.8	249.6	3.5	32.8
Total							164.1

**Table 2:** Average daytime monthly microclimatic data measured under (UP), between (BP) and outside the photovoltaic panels (T) during the growing season of medicinal crops (April–August 2023). Variables include air temperature (T_UP_, T_BP_ and T_T_, respectively), radiation (Rad_UP_, Rad_BP_ and Rad_T_) reference evapotranspiration (Et_0UP_, ET_0BP_ and ET_0T_), crop coefficients (Kc) and crop evapotranspiration (ETc_UP_, ETc_BP_ and ETc_T_).

Month	T_UP_	T_BP_	T_T_	Rad_UP_	Rad_BP_	Rad_T_	ETo_UP_	ETo_BP_	ETo_T_	Kc	ETc_UP_	ETc_BP_	ETc_T_
	(°C)	(°C)	(°C)	(Wm^−2^)	(Wm^−2^)	(Wm^−2^)	(mm)	(mm)	(mm)		(mm)	(mm)	(mm)
April	14.8	14.9	15.0	50	450	517	12.1	59.3	67.5	0.40	4.8	23.7	27.0
May	22.4	22.6	22.7	70	478	549	17.1	75.4	85.5	0.60	10.3	45.2	51.0
June	28.9	29.1	29.3	110	644	740	25.1	109.0	124.1	0.80	20.1	87.2	99.2
July	29.5	29.8	29.9	115	740	796	25.9	125.0	134.3	0.95	24.6	118.7	127.6
August	28.0	28.4	28.4	80	616	708	20.1	92.9	118.0	0.95	19.1	87.4	112.1
Mean	24.7	25.0	25.1	85	586	662				0.74			
Total							281.2	461.6	529.4		78.9	362.2	416.9

## Data Availability

Data are contained within the article.
